# Fine-scale thermal adaptation in a green turtle nesting population

**DOI:** 10.1098/rspb.2011.1238

**Published:** 2011-09-21

**Authors:** Sam B. Weber, Annette C. Broderick, Ton G. G. Groothuis, Jacqui Ellick, Brendan J. Godley, Jonathan D. Blount

**Affiliations:** 1 Centre for Ecology and Conservation, College of Life and Environmental Sciences, University of Exeter, Cornwall Campus, Penryn, UK; 2 Behavioural Biology, Centre for Behaviour and Neuroscience, University of Groningen, Groningen, The Netherlands; 3 Ascension Island Turtle Group, Georgetown, Ascension Island ASCN 1ZZ, UK

**Keywords:** local adaptation, phenotypic plasticity, climate change, natal homing, population structure, evolutionary significant unit

## Abstract

The effect of climate warming on the reproductive success of ectothermic animals is currently a subject of major conservation concern. However, for many threatened species, we still know surprisingly little about the extent of naturally occurring adaptive variation in heat-tolerance. Here, we show that the thermal tolerances of green turtle (*Chelonia mydas*) embryos in a single, island-breeding population have diverged in response to the contrasting incubation temperatures of nesting beaches just a few kilometres apart. In natural nests and in a common-garden rearing experiment, the offspring of females nesting on a naturally hot (black sand) beach survived better and grew larger at hot incubation temperatures compared with the offspring of females nesting on a cooler (pale sand) beach nearby. These differences were owing to shallower thermal reaction norms in the hot beach population, rather than shifts in thermal optima, and could not be explained by egg-mediated maternal effects. Our results suggest that marine turtle nesting behaviour can drive adaptive differentiation at remarkably fine spatial scales, and have important implications for how we define conservation units for protection. In particular, previous studies may have underestimated the extent of adaptive structuring in marine turtle populations that may significantly affect their capacity to respond to environmental change.

## 1. Introduction

In spatially heterogeneous environments, local populations exposed to contrasting selective regimes may diverge for traits that affect survival and reproduction [1]. Quantifying adaptive differentiation in animal populations has long been a cornerstone of ecology and evolutionary biology [1], and is becoming increasingly relevant to conservationists as a means of identifying evolutionarily significant units for protection [2]. Until recently, efforts to define population structure and management units in endangered species have tended to focus on ‘neutral’ genetic markers, which often underestimate the extent of adaptive differentiation in traits under strong selection [3]. Yet, these may be the very traits that most affect the ability of populations to respond to environmental change. For example, given future climate change predictions [4], the extent of adaptive variation in heat-tolerance is likely to have important consequences for the resilience of many ectothermic species in a rapidly warming world [5–9]. This has led to calls for an ‘adaptive evolutionary’ approach to conservation, which seeks to conserve functional diversity (rather than simply genetic marker diversity) at whatever scale it occurs [2].

Migratory species often present particular problems for conservationists when defining relevant spatial units for protection [10]. Given that local adaptation is opposed by gene flow and disrupted in temporally variable environments [1], species that migrate widely across a variety of different habitats might be expected to exhibit relatively low levels of adaptive differentiation. However, in some cases, the tendency for migrants to return to their own natal sites to reproduce has driven local adaptation at surprisingly fine spatial scales (e.g. in fishes [9,11–13]). For example, recent studies in anadromous salmon have shown that the thermal physiology of both adults and developing embryos are adapted to temperatures experienced in their natal spawning rivers [9,13]. Such studies have been instrumental in defining management units that conserve the evolutionary heritage and adaptive potential of salmonid populations [14], but are lacking for many other migratory species. Like salmonids, marine turtles are renowned for migrating long distances to breed at their natal nesting beaches [15], and have become flagship species for conservation initiatives [16]. However, to date, assessments of nesting population structure and designation of management units in these species have largely been based on molecular genetic approaches [17,18], with almost nothing known about the extent of adaptive phenotypic variation in thermal-tolerance, or any other trait. This is surprising, given that temperature has a profound effect on hatching success, embryonic development and sex in marine turtles [19,20], leading to growing concerns regarding the impacts of climate warming on their reproductive success [16,21,22].

**Figure 1.**
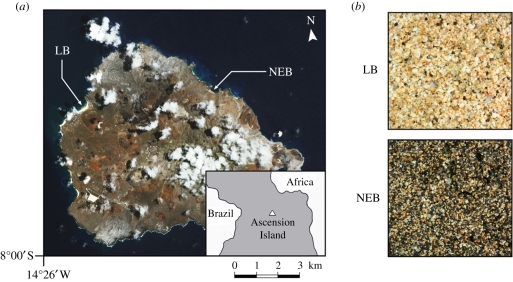
Overview of study sites. (*a*) Satellite image and location map (inset) of Ascension Island showing the two major green turtle nesting beaches used in the study (LB: Long Beach, NEB: Northeast Bay; image courtesy of NASA). (*b*) Sand sampled from LB and NEB photographed in the laboratory under standardized lighting and exposure to illustrate differences in colour.

Here, we present the results of a study to test for local adaptation in an island-nesting population of green turtles (*Chelonia mydas*) where incubation temperatures vary dramatically among closely adjacent nesting beaches. Ascension Island in the South Atlantic Ocean hosts one of the world's largest breeding populations of green turtles [23] (figure 1), comprising migrants from feeding grounds along the coast of Brazil over 2000 km to the west [24]. Nesting at Ascension Island occurs on both biogenic (pale sand) and volcanic (dark sand) beaches in close proximity to one another [25] (figure 1). Dark sand absorbs more solar radiation than pale sand leading to significant heterogeneity in incubation temperatures among nesting sites despite the island's small size [25]. For example, Ascension's two primary nesting beaches, Long Beach (LB: pale sand) and Northeast Bay (NEB: dark sand) differ in sand temperatures by a consistent 2–3°C despite being just 6 km apart [25] (figure 1). While all females could potentially nest at any site, individuals apparently discriminate between LB and NEB with a majority using the same beach within and among years [26]. Population genetic studies have established that most (if not all) females nesting at Ascension Island hatched at this site themselves [27,28], and subsequent work has revealed weak, but significant matrilineal divergence between NEB and LB that might suggest precise natal homing [29,30]. Although the genetic differences are slight (*F*_ST_ = 0.042) [29], similar levels of differentiation at neutral loci are often associated with significant divergence in traits under selection [3]. Consequently, we hypothesized that the thermal tolerances of developing turtle embryos may have diverged and adapted to the contrasting thermal regimes at these nesting sites.

Local adaptation is often assessed by comparing the relative fitness of local versus foreign genotypes under common-garden conditions [1]. This requires relevant measures of both fitness and also maternal effects, as the latter may mimic local adaptation if females adjust offspring phenotypes to suit specific developmental environments [1]. However, such maternal effects have rarely been comprehensively studied (e.g. [6]). We used a combination of *in situ* and common-garden approaches to compare survival (as a measure of fitness), developmental rates and size at hatching for offspring of LB and NEB females at different incubation temperatures, while simultaneously accounting for egg-mediated maternal effects. Detailed biochemical assays were conducted for all egg components that might be expected to modify embryonic thermal tolerances and growth trajectories. These included egg mass and energetic resources that have been linked to temperature-tolerance in birds [31], yolk fatty acid profiles that may modify the thermal stability of lipid membranes [32], and maternally derived antioxidants and steroid hormones that may affect the ability to cope with thermally induced oxidative stress [33] and accelerate embryonic metabolism and growth rates, respectively [34].

## 2. Material and methods

### (a) Study site

Ascension Island (UK) is an isolated volcanic peak in the South Atlantic Ocean (figure 1). Green turtle nesting generally occurs between January and June, with a majority of the activity focused at a small number of primary nesting sites [35]. LB and NEB support the highest numbers and densities of nesting females on the island (36% and 12% of total nests, respectively [35]), but provide very different developmental environments: NEB is composed of black volcanic sand and is on average 2.6°C hotter at nest depth (approx. 75 cm) than LB which has paler sand with a high proportion of biogenic material [36]. Intra-beach variation in sand temperatures is low compared with variation between beaches [25,36], and inter-annual variability is minimal, with differences in sand temperature between LB and NEB thought to have remained constant over long (generational) timescales [36,37].

### (b) Temperature and hatching success *in situ*

In 2004, an observational study was undertaken to relate the hatching success of *in situ* clutches to nest temperatures at LB and NEB, and to obtain baseline data on normal ranges of incubation temperatures at these sites. Females nesting at LB and NEB were selected at random between 22 February and 7 March 2004 (*n* = 30 per beach). An archival temperature logger (Tinytalk, Gemini Data Loggers, Chichester, UK; precision of 0.3°C) was placed at the centre of each clutch during oviposition and was programmed to record nest temperature at 4 h intervals throughout the incubation period. These data were then used to calculate mean incubation temperatures for each clutch. Loggers were recovered during nest excavation following hatchling emergence, and hatching success was estimated from the number of hatched and unhatched eggs.

### (c) Common-garden incubation experiment

In 2008, we conducted a common-garden experiment, using artificial incubators to approximate the temperatures of *in situ* nests on LB and NEB. Artificial incubation was carried out in custom-built forced air incubators, constructed of expanded polystyrene and controlled by pulse-proportional thermostats (HabiStat; Euro Rep, Hayes, UK). Temperatures were set at either a constant 32.5°C (‘hot treatment’) or a constant 29°C (‘cool treatment’), values, which are within the range of incubation temperatures for *in situ* nests at NEB and LB, respectively (see §3). Internal incubator temperatures were monitored continuously using Tinytalk dataloggers (cross-calibrated against an NIST-certified mercury thermometer) and remained within ±0.3°C of nominal values throughout incubation.

Carrying out *ex situ* incubation and analyses of eggs in an endangered species require careful justification. We obtained permission to collect eggs from *n* = 10 clutches per beach for females nesting at LB and NEB between 18 and 28 March 2008. This corresponds with the period of peak nesting activity at Ascension Island. [35]. Nine eggs were sampled during oviposition from near to the start (three eggs), middle (three eggs) and end (three eggs) of the laying sequence for each clutch (mean ± s.e. clutch size in this population is 124 ± 4 eggs). Within 1 h of oviposition, two eggs from each laying sequence position were allocated at random to artificial incubation: one egg to the hot incubation treatment, and one egg to the cool treatment. The remaining sampled eggs were used for biochemical analyses (see later text).

Eggs were incubated half buried in moist vermiculite hydrated to constant water potential of approximately –50 kPa throughout incubation [38] in individual plastic containers sealed with loosely fitting lids to maintain humidity, and perforated with a standard number of holes to permit gas exchange. Open trays of water were also placed inside incubators to create a humid atmosphere, and incubators were aerated by opening for 5 min daily. Incubators were closely monitored to determine hatching dates, and eggs that failed to hatch were dissected to determine the stage of embryonic mortality (all eggs were fertile as evidenced by ‘chalking’ on the upper shell surface at the site of vitelline membrane attachment [38]). Staging of marine turtle embryos conventionally follows the 31 stage system proposed by Miller [39]. However, in the present study, embryos were either too small to stage by the naked eye, or were morphologically fully formed when they died (approx. stages 27–29 in Miller [39]). Since stages 27–29 differ primarily with respect to embryo size [39], we expressed the stage of mortality as the mass of embryos as a percentage of total egg contents. Within approximately 24 h of emergence, all surviving hatchlings were weighed (±0.2 g), and straight carapace length (SCL) was measured using a digital calliper (±0.1 mm). By this time, hatchling carapaces had fully straightened and residual yolks had been internalized.

### (d) Analyses of egg composition

Three eggs were sampled for compositional analysis from each experimental clutch (as above). Within 1 h of collection, eggs were carefully separated into their constituent parts (albumen, yolk) and weighed. The yolk portion was homogenized and both fractions stored at −45°C awaiting biochemical analysis. Water content of yolk and albumen was calculated gravimetrically following lyophilization of pre-weighed samples to a constant mass. Total lipids were extracted from an aliquot of yolk by homogenization in chloroform/methanol (2 : 1, v/v) and lipid content determined gravimetrically after evaporation of the solvent [40]. The fatty acid composition of a portion of the lipid extract was then analysed by gas chromatography/mass spectrometry following derivitization to form fatty acid methyl esters (FAMEs) as described previously [41]. FAMEs were separated using a TraceMS instrument (ThermoQuest, Hemel Hempstead, UK) fitted with a Factor Four VF23-MS-fused silica capillary column (high cyanopropyl-modified methyl polysiloxane; Varian Chrompack, 60 m × 0.32 mm, 0.15 µm film thickness). A two-step temperature programme was used from 40°C (held for 2 min) to 100°C at 13°C min^−1^, and then at 3°C min^−1^ to 260°C (held for 10 min) with helium as the carrier gas (flow rate is 2 ml min^−1^). Peaks were identified by comparison with the retention times of standard FAME mixtures (Supelco, Bellefonte, PA, USA) and peak areas integrated to express amounts of individual compounds as a proportion of total fatty acids. Vitamin E concentrations in yolk were assayed following alkaline saponification to remove bulk lipids, and quantified using high-performance liquid chromatography (HPLC) calibrated with standard solutions of α- and γ-tocopherol (Sigma-Aldrich, St Louis, MO, USA) as described previously [42]. Carotenoids were extracted from egg yolk and quantified using HPLC following previously described methods [42]. Concentrations of maternally derived testosterone and oestradiol in egg yolk were analysed after extraction with petroleum ether/diethylether and methanol, and quantified using radio-immuno assays with commercially available kits (see the study of Casagrande *et al*. [43] for details).

### (e) Statistical analysis

The hatching success of *in situ* clutches (binomial variable: number hatched/number unhatched) was modelled as a function of mean incubation temperature using generalized linear modelling (GLM) with a quasi-binomial error structure (to account for binomial overdispersion) and with beach of origin included as a fixed factor. Significance of the explanatory variables was evaluated using type III *F*-tests following deletion from the model, starting with the temperature × origin interaction. As temperatures in marine turtle nests increase progressively over the incubation period [44], we also performed an analysis relating *in situ* hatching success to maximum nest temperatures. However, the results of this analysis were qualitatively the same as those for mean incubation temperature and are not discussed further. The hatching success of eggs in the common-garden experiment was analysed using generalized linear mixed modelling (GLMM) with treatment and origin as fixed factors, clutch included as a random factor, and the arcsine square-root transformed proportion of eggs hatched from each clutch within a treatment as a response variable. Hatchling size (SCL) and residual body mass (controlling for SCL) were analysed using GLMM with treatment and origin as fixed factors and clutch included as a random factor. Since eggs in the common-garden experiment were sampled at three points during the laying sequence of each clutch (see earlier text), laying sequence position and its two-way interactions with origin and temperature were initially included in all analyses of hatchling phenotypes, but were always non-significant (*p* > 0.30) and so are not discussed further. Significance of fixed effects in GLMMs was assessed using likelihood-ratio tests (compared against a *χ*^2^-distribution) following deletion from the model, starting with the temperature × origin interaction (α = 0.05). Compositional measurements of eggs laid by females nesting at NEB and LB were compared using unpaired *t*-tests on clutch means (*n* = 3 eggs per clutch), with the exception of yolk fatty acid profiles that were first reduced to a smaller number of uncorrelated variables using a principal component analysis (PCA) on centred log-ratio transformed data [45]. Principal component (PC) scores on the major axes (eigenvalues greater than 1) were then compared by multivariate analysis of variance (MANOVA). Data are presented as means ± 1 s.e., and all analyses were carried out using R v. 2.9.2 [46].

## 3. Results

**Figure 2.**
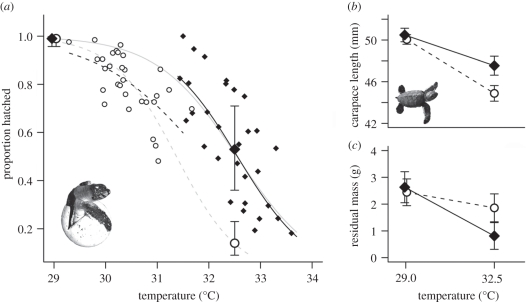
Effects of incubation temperature on hatching success and hatchling morphology for clutches laid at Long Beach (LB: open circles, dashed lines) and Northeast Bay (NEB: filled diamonds, solid lines). (*a*) Small symbols show hatching success of *in situ* nests across a natural range of incubation temperatures with lines in bold fitted using logistic regression. Large symbols show mean hatching success from a common-garden experiment where eggs were incubated at either 29°C (‘cool’) or 32.5°C (‘hot’). Values are binomial estimates ± 1 s.e. Faded lines showing overall temperature–survival curves were fitted using logistic regression with data from *in situ* nests and constrained to pass through estimates from the common-garden experiment. Parts (*b*,*c*) show effects of incubation temperature and beach of origin on hatchling straight carapace length (SCL) and residual body mass (controlling for SCL), respectively, in the common-garden experiment (estimates ± 1 s.e. from GLMM).

Mean incubation temperatures differed significantly between *in situ* clutches laid on NEB and LB. Nests on NEB were on average 2.2°C hotter and there was very little overlap in temperatures experienced between sites (NEB: mean = 32.4 ± 0.1°C, range = 31.5–33.6; LB: mean = 30.2 ± 0.1°C, range = 29.2–31.6). The proportion of eggs hatching decreased at higher incubation temperatures at both beaches (GLM: temperature, *F*_1,58_ = 56.2, *p* < 0.001); however clutches laid at NEB had higher hatching success at a given temperature compared with LB clutches, suggesting an increased upper thermal-tolerance limit for the offspring of NEB females (origin, *F*_1,58_ = 9.3, *p* = 0.004; temperature × origin, *F*_1,57_ = 1.2, *p* = 0.27; figure 2*a*).

**Figure 3.**
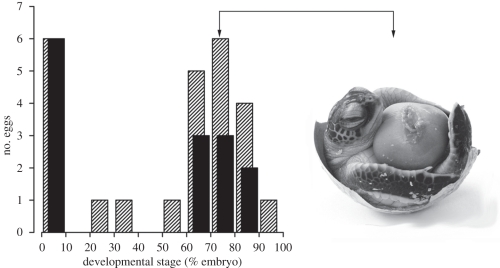
Stages of embryonic mortality for unhatched eggs from LB and NEB in the hot incubation treatment (32.5°C). Developmental stage is expressed as the mass of the yolk free embryo as a proportion of total egg contents (i.e. embryo + yolk sac). The photograph shows a representative embryo at 70–80% development (‘late-stage’ mortality) with yolk sac attached. Striped bars, LB; black bars, NEB.

This interpretation is supported by the results of the common-garden rearing experiment. While hatching success was equally high for eggs from both beaches in the cool incubation treatment (97% of eggs hatched in each case), eggs laid by females nesting on NEB had markedly improved hatching success in the hot treatment compared with those laid by LB females (proportion eggs hatched, NEB: 53%, LB: 17%; figure 2*a*). This resulted in a significant temperature × origin interaction for hatching success (GLMM: temperature, 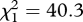, *p* < 0.001; origin, 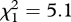, *p* = 0.024; temperature × origin, 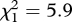, *p* = 0.015; figure 2*a*). Eggs in the hot treatment that did not hatch were dissected to determine the stage at failure (data for the cool treatment are not presented as all but one egg from each beach of origin hatched). Stages of embryonic mortality were bimodally distributed with either no visible embryos (only blood spots and/or shell chalking apparent: ‘early-stage’ mortality), or large, fully formed embryos present (embryo mass greater than 20% of total egg contents: ‘late-stage’ mortality; figure 3). The proportion of eggs containing early-stage dead embryos was identical for both beaches (20% in each case; binomial GLMM: 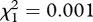, *p* = 0.98); so the disparity in hatching success that we observed in the hot treatment was owing to a significant increase in late-stage embryonic mortality for eggs laid by females nesting at LB compared with NEB females (NEB: 27%, LB: 63%; binomial GLMM 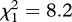, *p* = 0.004; figure 3).

In addition to effects on embryo survival, we also examined how different incubation regimes influenced development times and hatchling morphology. Eggs developed faster in the hot treatment (days to hatching, hot: mean = 43.9 ± 0.2 d, cool: mean = 53.8 ± 0.2 d), as is typical in reptiles [20], but development times were comparable for eggs laid at LB and NEB (GLMM: temperature, 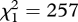, *p* < 0.001; origin, 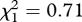, *p* = 0.40; temperature × origin, 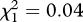, *p* = 0.84). However, results for hatchling morphology mirrored those found for hatching success. SCL and residual body mass (controlling for SCL) were similar for hatchlings from both beaches of origin in the cool treatment, but NEB hatchlings were significantly larger and lighter for their size than LB hatchlings in the hot treatment (GLMM, SCL: temperature, 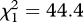, *p* < 0.001; origin, 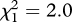, *p* = 0.16; temperature × origin, 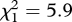, *p* = 0.015; residual body mass: temperature, 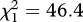, *p* < 0.001; origin, 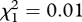, *p* = 0.92; temperature × origin, 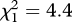, *p* = 0.035; figure 2*b,c*), indicating that growth trajectories of LB and NEB embryos were differentially affected by high incubation temperatures. Although we did not determine hatchling sex in this study, previous work indicates that hatchlings from NEB and LB are exclusively female at an incubation temperature of 32.5°C [47].

As an alternative to local adaptation, we also considered the possibility that embryonic thermal tolerances may be influenced by non-genetic maternal effects mediated through resource-provisioning in eggs. However, we found no evidence to support this. Clutches laid at LB and NEB did not differ significantly in terms of masses of eggs, yolk, albumen, water and lipid, or yolk concentrations of vitamin E and carotenoids (maternally derived antioxidants), testosterone and oestradiol (maternally derived steroids) or arcsine square-root-transformed proportions of saturated, polyunsaturated and monounsaturated fatty acids (unpaired *t*-tests, range of *t* = 0.004–1.35, range of *p* = 0.19–0.99; see electronic supplementary material, table S1). PCA of yolk fatty acid profiles yielded similar results. PC scores on the major axes (PC1: 32%, PC2: 24%, PC3: 13%) did not differ significantly for clutches laid at NEB and LB (MANOVA, *F*_3,16_ = 0.99, *p* = 0.42; electronic supplementary material, table S1), indicating that yolk fatty acid compositions were comparable for eggs from both beaches of origin.

## 4. Discussion

The results of this study suggest that the thermal tolerances of green turtle embryos at Ascension Island have diverged in response to the contrasting incubation temperatures of black- and white sand nesting beaches just a few kilometres apart (figures 1 and 2). In natural nests and in a common-garden rearing experiment, the offspring of females nesting at NEB had a higher probability of surviving to hatching (a direct measure of fitness) and grew larger (a potential determinant of early survival [48]) at hot incubation temperatures compared with the offspring of females nesting at LB (figure 2). This difference was owing to an expansion in the range of temperatures that could be tolerated by NEB turtles rather than a shift in the thermal optimum. Offspring size and survival decreased at higher incubation temperatures in both populations, but the slopes of the thermal reaction norms for these traits were significantly shallower for turtles nesting at NEB (figure 2). Non-genetic maternal effects are unlikely to explain this finding, as a detailed analysis of egg composition revealed no significant differences between sites (see the electronic supplementary material). Rather, the results of the common-garden experiment suggest that the increased heat-tolerance of NEB turtles has a genetic basis, and is adaptive, resulting in smaller reductions in embryo survival and hatchling size at the high incubation temperatures that prevail at this site (figure 2).

To our knowledge, this is the first study to demonstrate local adaptation in a marine turtle population. A previous study suggested that the post-hatching migratory behaviour of Florida green turtles may have diverged between east and west coast populations, but the authors were unable to rule out alternative explanations for their results or relate them to fitness [49]. The patterns of divergence that we document are particularly surprising given the close proximity of LB and NEB (6 km versus a 2000 km maternal migration; figure 1), and the almost unlimited potential for gene flow between these sites if females were to nest randomly. Strong selection against less heat-tolerant genotypes at NEB may limit effective levels of gene flow (e.g. [11]), but this cannot account for the superior heat-tolerance of NEB embryos in the present study, which was determined using eggs collected directly from nesting females (i.e. before any mortality occurred). Rather, our results suggest that, as in salmon [9,11,13], the tendency for female sea turtles to nest at their own natal sites may have driven and maintained the observed divergence in thermal-tolerance. Population genetic studies have revealed weak (but significant) structuring between females nesting at NEB and LB, offering limited support for precise natal homing [29,30]. However, given the small amount of gene flow needed to disrupt divergence at selectively neutral loci [3] and the exceptionally slow rate of genetic drift in sea turtles [27], detecting even weak divergence at neutral markers between these two closely adjacent nesting sites is surprising. Indeed, behavioural studies have shown that while the majority of females return faithfully to the same nesting site within and among years, some ‘straying’ also occurs [26]. Such movements are likely to severely restrict differentiation at neutral loci, whereas traits under strong selection (such as thermal-tolerance) may diverge despite some gene flow between populations [3,12].

In contrast to the strong site fidelity of females, male green turtles at Ascension Island are thought to mate indiscriminately, leading to significant male-mediated gene flow among nesting sites [30] (see Bowen & Karl [17] for a general review of male-mediated gene flow in marine turtles). In fact, because sex is temperature-dependent in marine turtles and incubation temperatures at NEB are predominantly feminizing [47], we can be confident that most NEB females will mate with males hatched on an adjacent cooler beach. This should severely disrupt divergence in all but maternally inherited traits, raising the intriguing possibility that thermal-tolerances have diverged along maternal lineages via mitochondrial genes (as sea turtles lack distinct sex chromosomes). Although molecular studies are needed to test this hypothesis, there are sound theoretical reasons to suggest that thermal-tolerance could be inherited mitochondrially.

According to the oxygen-limited thermal-tolerance hypothesis [33], the ability to maintain aerobic scope at different temperatures and rates of metabolism are fundamental in determining the thermal limits to growth and survival (e.g. [9]). The mitochondria play a key role in setting oxygen-limited thermal-tolerance windows [33] and their genomes have been identified as targets of selection in thermally heterogenous environments [50]. Indeed, while we can only speculate on the underlying mechanisms at present, several aspects of our results suggest that an adaptive increase in aerobic scope may explain the superior performance of NEB embryos in hotter environments. First, significant differences in survival between NEB and LB embryos in the hot incubation treatment were only apparent late in development (stages 27–29 in Miller [39]; figure 3), when rates of growth and oxygen consumption are maximal [51]. Thus, high temperatures were not lethal for LB embryos *per se*, but became limiting during the phase of maximum aerobic demand. Second, oxygen-deprived reptilian embryos typically hatch smaller with larger residual yolk sacs owing to metabolic depression and impaired yolk use [52]; so the reduced size and increased residual body mass of LB hatchlings in the hot incubation treatment are consistent with oxygen-limited growth compared with locally adapted NEB hatchlings (figure 2*c*). Further work using respirometry to compare the aerobic performance of embryos from different nesting beaches over a range of temperatures would be a logical next step in testing this mechanism.

In summary, this study has demonstrated significant adaptive differentiation in the thermal-tolerance of green turtle embryos between two closely adjacent nesting beaches. The fine spatial scale at which divergence has occurred despite the absence of geographical barriers to gene flow is particularly remarkable, and has probably been driven by the tendency for female sea turtles to nest in their own natal environments [15,28], as in salmon [9,11,13] and some brood parasitic birds (e.g. [53]). Moreover, these findings highlight the potential for adaptive divergence among more geographically isolated marine turtle populations, and thus have important implications for how we define evolutionarily significant units for conservation. As in many species of conservation concern, attempts to define population structure and conservation units in sea turtles have largely been limited to molecular approaches using ‘neutral’ genetic markers [17,18], and have generally revealed only regional differentiation between nesting populations [17]. Our results suggest that management strategies may need to consider adaptive population structuring at finer spatial scales than is often implied by genetic markers if we are to effectively conserve the evolutionary potential of marine turtle populations (see also Fraser & Bernatchez [2] and McKay & Latta [3]).

Adaptive variation in heat-tolerance is likely to be particularly relevant to marine turtle conservation and resilience if it proves to be more widespread. Global warming is predicted to have multiple deleterious effects on the reproductive success of marine turtles, including the loss of nesting beaches to rising sea levels, increasingly feminized populations and reduced hatching success [16,21,22]. Our results suggest that in at least one of these respects, marine turtles have the capacity to adapt to warmer temperatures. However, the implications of this are currently unclear. Locally adapted populations may be more susceptible to extinction as they lack the flexibility to respond to warming temperatures [54]. However, migration of individuals from heat-adapted populations may confer a degree of resilience to the effects of climate change at broader spatial scales [7,8]. Future studies should explore whether other thermally sensitive traits, particularly sex determination, are locally adapted in marine turtles, and attempt to integrate these findings into models predicting population responses to climate change (e.g. [22]). Given the long-generation times of sea turtles, it is also unclear whether thermal adaptation could keep pace with the rapid warming predicted by climatic models [4]. Indeed, although this study suggests that NEB embryos have adapted to hotter incubation temperatures, it is worth noting that hatching success at this site is still significantly below that found at LB (figure 2). While there could be a number of reasons for this, climate reconstructions for Ascension Island indicate a 0.5°C rise in average nesting beach temperatures over the past 150 years (approx. four to six green turtle generations) [37]. Based on contemporary thermal performance curves, this suggests that mean hatching success at NEB may have historically stood at 72 per cent, close to present-day levels at LB (mean = 80%; figure 2*a*). Thus, the currently lower hatching success at NEB might reflect embryonic thermal tolerances already failing to evolve in step with anthropogenic climate change.
